# Supplementary material for: The impact of climate change on economic output across industries in Chile
Source: PLoS One. 2022 Apr 28;17(4):e0266811. doi: 10.1371/journal.pone.0266811 (PMC9049569; doi:10.1371/journal.pone.0266811)
Supplement: S1 Appendix — (PDF) [file pone.0266811.s001.pdf]

S1 Appendix. Panel-level heterogeneity and unit root tests

We implemented panel data tests for heterocedasticity and unit roots of the real growth rate of each industry ($\Delta y_{r,i,t}$), according to the methodologies suggested in (1; 2). By estimating an ordinary GLS panel data model of the growth of each industry i across different regions and time, we then perform a Likelihood Ratio Test of whether the regions are heterocedastic or not (2). Table A1 shows that the hypothesis of homocedasticity of the residuals is rejected by the Likelihood ratio test of the GLS model (LRT-GLS), whether we include year fixed-effects or a time trend. We also implement the Wooldridge test for serial autocorrelation in linear panel data models, using the code of (3). The p-values for the Wooldridge test (2) reported in Table A1 reject the null hypothesis of no autocorrelation. For this reason our option of including standard-errors clustered by region and time in the panel data regressions of section 3.1 is justified.

Table A1. P-values of the tests for heterocedasticity across regions and autocorrelation of the panel residuals of the real growth rate $\Delta y_{r,i,t}$

Industry / region	Panel heterocedasticity tests<br>LRT-GLS<br>with year fixed effects	LRT-GLS<br>with time trend	Autocorrelation<br>Wooldridge<br>test
Agriculture and Forestry	3.08E-08	1.57E-05	9.66E-06
Fishing	5.26E-06	6.02E-21	0.0149
Mining	6.06E-48	1.19E-25	3.31E-06
Manufacturing	1.48E-47	2.08E-31	1.36E-08
Electricity, Gas, and Water (EGA)	4.8E-30	2.32E-29	4.21E-06
Construction	3.77E-53	1.29E-30	1.10E-07
Commerce, Restaurants, and Hotels	0.0004	6.16E-09	6.89E-06
Transport and Communications	1.28E-13	5.18E-06	0.0004
Financial Services	3.92E-32	1.84E-06	1.90E-10
Home Ownership	1.6E-178	0.001	8.78E-06
Personal Services	1.33E-14	0.0299	8.83E-10
Public Administration	8.1E-18	0.0064	3.83E-12

Table A2 reports the panel data unit root tests suggested in (1) for the null hypothesis of a unit root in the real growth rate of each industry i ($\Delta y_{r,i,t}$): Levin–Lin–Chu (LLC), Harris–Tzavalis (HT), Breitung, Im–Pesaran–Shin (IPS), Fisher with Dickey Fuller option (Fisher-DF), Fisher with Phillips-Perron option (Fisher-PP). All the tests reject the hypothesis of unit-root in the real growth series of each industry. Furthermore, the Hadri stationarity test suggested in (1) does not reject the null hypothesis of stationarity for 8 industries, which again suggests that stationarity is the most fitting option for the econometric model of this article. For these reasons our option of assuming a stationary model for the linear panel data model of section 2.3 is justified.

Table A2. P-values of the panel unit root tests of the real growth rate of each industry i ($\Delta y_{r,i,t}$)

Industry / region	Tests with null hypothesis of unit root						Stationarity
	LLC	HT	Breitung	IPS	Fisher-DF	Fisher-PP	Hadri test
Agriculture and Forestry	1.49E-73	0.000	6.95E-16	5.89E-36	6.11E-40	6.11E-40	0.864
Fishing	5.18E-99	0.000	1.73E-24	1.29E-39	3.60E-99	3.60E-99	0.981
Mining	8.54E-27	1.79E-64	1.03E-06	3.26E-21	1.87E-33	1.87E-33	0.286
Manufacturing	2.77E-52	0.000	2.05E-13	2.31E-30	3.03E-42	3.03E-42	0.513
Electricity, Gas, and Water (EGA)	3.87E-40	3.50E-75	3.84E-23	9.61E-26	1.51E-40	1.51E-40	0.950
Construction	4.79E-66	0.000	3.30E-28	6.34E-34	1.69E-56	1.69E-56	0.982
Commerce, Restaurants, and Hotels	2.35E-37	1.14E-72	2.08E-18	4.93E-25	1.36E-19	1.36E-19	0.245
Transport and Communications	7.88E-52	5.22E-89	3.04E-10	9.13E-29	3.66E-49	3.66E-49	0.000
Financial Services	2.10E-71	0.0000	3.61E-22	5.39E-35	6.23E-36	6.23E-36	0.243
Home Ownership	1.82E-19	5.12E-28	8.80E-13	2.84E-15	8.18E-02	8.18E-02	0.000
Personal Services	4.79E-33	2.57E-50	2.80E-12	8.12E-22	4.03E-06	4.03E-06	0.000
Public Administration	6.17E-46	6.46E-71	2.76E-12	3.19E-26	9.10E-07	9.10E-07	0.000

References

1. Baltagi, B. (2021). "Econometric analysis of panel data". Springer Nature.
2. Wooldridge, J. (2010). "Econometric Analysis of Cross Section and Panel Data". MIT Press.
3. Drukker, D. (2003). "Testing for Serial Correlation in Linear Panel-Data Models". *Stata Journal*, 3(2), 168-177.
