# Supplementary material for: The impact of climate change on economic output across industries in Chile
Source: PLoS One. 2022 Apr 28;17(4):e0266811. doi: 10.1371/journal.pone.0266811 (PMC9049569; doi:10.1371/journal.pone.0266811)
Supplement: S2 Appendix — This appendix show some robustness checks using the same model of industry-region GDP with temperature and precipitation fluctuations with constant weights for each industry and different clustering options (clusters just by year or clusters by region-year). (PDF) [file pone.0266811.s002.pdf]

S2 Appendix. Other model estimates

As a robustness check, Table B1, Table B2 and Table B3 estimate the same model of industry-region GDP with temperature and precipitation fluctuations using constant weights for each industry and different clustering options (clusters just by year or clusters by region-year). Table B1 shows the model with quarterly weather fluctuations and 15 regions. Table B2 and Table B3 also aggregate regions I and XV plus regions X and XIV, therefore presenting 13 regions for the entire period of 1985 to 2017. Table B2 presents the coefficients for the model with quarterly weather fluctuations and 13 regions. Table B3 presents the coefficients for the model with monthly weather fluctuations and 13 regions.

Table B1. Coefficients for the impact of temperature and precipitation (quarterly averages) on regional industry GDP

	Agriculture	Fishing	Mining	Manufact.	EGA	Constr.	Commerce	Transp.	Finan. serv.	Home	Pers. serv.	Pub. adm.
	(1)	(2)	(3)	(4)	(5)	(6)	(7)	(8)	(9)	(10)	(11)	(12)
Coefficients for Temperature												
Jan-Mar	-0.031**	-0.087	0.045**	0.006	-0.037	-0.012	0.007	0.007	0.007	0.001	-0.002	0.002
	(0.013)	(0.061)	(0.019)	(0.010)	(0.027)	(0.012)	(0.011)	(0.005)	(0.005)	(0.002)	(0.002)	(0.002)
Apr-Jun	0.025*	0.076*	-0.069**	0.025***	0.038	0.002	-0.007	-0.002	-0.013*	0.001	-0.004*	0.003
	(0.014)	(0.044)	(0.033)	(0.007)	(0.029)	(0.016)	(0.009)	(0.006)	(0.007)	(0.002)	(0.002)	(0.003)
Jul-Sep	-0.020	-0.002	-0.003	-0.013	0.012	-0.005	0.008	0.002	0.010*	-0.004	-0.001	-0.004
	(0.014)	(0.045)	(0.020)	(0.013)	(0.028)	(0.017)	(0.007)	(0.005)	(0.005)	(0.005)	(0.004)	(0.003)
Oct-Dec	0.023	-0.022	0.045**	0.019*	0.019	-0.014	-0.001	0.002	0.004	-0.003	0.006	-0.004
	(0.018)	(0.037)	(0.019)	(0.010)	(0.028)	(0.020)	(0.005)	(0.008)	(0.009)	(0.003)	(0.004)	(0.003)
Coefficients for Precipitation												
Jan-Mar	0.002	0.007	-0.013	0.010***	0.016	-0.007	-0.002	-0.001	-0.002	-0.001	0.002**	-0.001**
	(0.005)	(0.015)	(0.019)	(0.003)	(0.011)	(0.012)	(0.003)	(0.002)	(0.002)	(0.001)	(0.001)	(0.001)
Apr-Jun	0.003	-0.017*	-0.002	-0.000	0.013*	-0.004	-0.001	-0.000	-0.000	0.000	0.000	-0.000
	(0.002)	(0.010)	(0.004)	(0.001)	(0.007)	(0.004)	(0.001)	(0.001)	(0.001)	(0.001)	(0.000)	(0.000)
Jul-Sep	-0.002	0.015	0.004	0.004	0.014**	0.005	0.002	0.002	0.002***	0.001	0.001	-0.001
	(0.003)	(0.010)	(0.004)	(0.004)	(0.006)	(0.007)	(0.002)	(0.002)	(0.001)	(0.001)	(0.001)	(0.000)
Oct-Dec	0.000	-0.013	0.030***	-0.005**	0.007	0.011	0.000	-0.003	-0.000	0.000	0.002*	-0.001
	(0.004)	(0.012)	(0.008)	(0.003)	(0.005)	(0.012)	(0.003)	(0.002)	(0.002)	(0.001)	(0.001)	(0.001)
N	465	436	395	465	460	465	465	465	465	465	465	465
R^2	0.104	0.137	0.062	0.059	0.058	0.026	0.023	0.087	0.069	0.131	0.055	0.067

Notes: Observations weighted by constant regional industry GDP share. Lagged industry GDP growth included in each regression. Lagged industry GDP growth included in each regression. Robust standard errors clustered by year and region in parentheses. ** p<0.01, ** p<0.05, * p<0.1

Table B2. Coefficients for the impact of temperature and precipitation (quarterly averages) on the regional industry GDP for 13 regions

	Agriculture (1)	Fishing (2)	Mining (3)	Manufact. (4)	EGA (5)	Constr. (6)	Commerce (7)	Transp. (8)	Finan. serv. (9)	Home (10)	Pers. serv. (11)	Pub. adm. (12)
Coefficients for Temperature												
Jan-Mar	-0.021*	-0.047	0.060**	0.009	-0.033*	-0.017	-0.007	0.006	0.004	-0.003	0.001	0.000
	(0.011)	(0.035)	(0.028)	(0.016)	(0.019)	(0.019)	(0.005)	(0.006)	(0.003)	(0.003)	(0.003)	(0.002)
Apr-Jun	0.014	0.011	-0.077**	0.015	0.027**	0.016	0.002	-0.007	-0.004	0.001	-0.004	0.003
	(0.012)	(0.033)	(0.039)	(0.010)	(0.013)	(0.014)	(0.005)	(0.006)	(0.005)	(0.002)	(0.003)	(0.002)
Jul-Sep	-0.015	0.027	0.088*	-0.017*	0.014	0.015	0.003	-0.001	0.007**	-0.004	0.003	-0.003
	(0.013)	(0.041)	(0.050)	(0.010)	(0.019)	(0.020)	(0.004)	(0.007)	(0.004)	(0.004)	(0.003)	(0.002)
Oct-Dec	0.023	0.007	-0.069	0.024*	0.003	-0.027	0.005	0.005	-0.002	-0.002	0.003*	-0.003
	(0.018)	(0.043)	(0.043)	(0.015)	(0.025)	(0.021)	(0.006)	(0.006)	(0.007)	(0.002)	(0.002)	(0.003)
Coefficients for Precipitation												
Jan-Mar	0.003	0.005	-0.004	0.011*	0.006	-0.007	-0.003	-0.003**	-0.002	-0.000	0.001	-0.000
	(0.005)	(0.011)	(0.014)	(0.006)	(0.008)	(0.013)	(0.003)	(0.002)	(0.002)	(0.000)	(0.001)	(0.001)
Apr-Jun	0.002	-0.017***	-0.005	-0.002	0.011*	-0.004	0.000	0.000	-0.000	0.000	0.000	0.000
	(0.003)	(0.006)	(0.008)	(0.002)	(0.005)	(0.004)	(0.000)	(0.001)	(0.001)	(0.001)	(0.001)	(0.000)
Jul-Sep	0.001	0.035***	0.003	0.004	0.008*	0.009*	-0.000	-0.000	0.002*	0.001	0.001	-0.001
	(0.003)	(0.008)	(0.010)	(0.005)	(0.005)	(0.005)	(0.002)	(0.002)	(0.001)	(0.001)	(0.001)	(0.001)
Oct-Dec	0.001	0.018	-0.020*	-0.004**	0.006	0.018**	0.001	-0.005*	-0.001	-0.000	0.000	-0.001
	(0.004)	(0.012)	(0.011)	(0.002)	(0.004)	(0.009)	(0.002)	(0.003)	(0.001)	(0.000)	(0.001)	(0.001)

Notes: Observations not weighted for GDP in regressions. Lagged industry GDP growth included in each regression. Regions 1 and 14, and regions 10 and 15 were merged for a total of 13 regions instead of 15 regions. Robust standard errors clustered by year and region in parentheses. ** p<0.01, ** p<0.05, * p<0.1

Table B3. Coefficients for the impact of temperature (monthly averages) on the regional industry GDP for 13 regions

	Agriculture	Fishing	Mining	Manufact.	EGA	Constr.	Commerce	Transp.	Finan. serv.	Home	Pers. serv.	Pub. adm.
	(1)	(2)	(3)	(4)	(5)	(6)	(7)	(8)	(9)	(10)	(11)	(12)
Jan	-0.035***	-0.074***	0.038*	-0.003	-0.044***	-0.012	-0.005	0.011*	0.000	-0.001	0.001	-0.003**
	(0.011)	(0.012)	(0.022)	(0.015)	(0.015)	(0.012)	(0.003)	(0.005)	(0.003)	(0.001)	(0.001)	(0.001)
Feb	0.007	0.028*	0.011***	-0.001	0.001	-0.024	0.003	0.002	-0.004	0.000	0.001	0.001
	(0.007)	(0.016)	(0.003)	(0.010)	(0.002)	(0.024)	(0.003)	(0.005)	(0.004)	(0.000)	(0.001)	(0.002)
Mar	0.008	0.007	0.001	0.025*	-0.005	0.014*	-0.001	-0.007	0.010***	-0.005	-0.000	0.003
	(0.011)	(0.043)	(0.035)	(0.013)	(0.014)	(0.008)	(0.005)	(0.006)	(0.004)	(0.004)	(0.002)	(0.002)
Apr	0.011	-0.016	-0.027	-0.005	0.038**	0.022	-0.003	0.000	0.001	-0.000	-0.006***	0.001
	(0.009)	(0.053)	(0.031)	(0.005)	(0.018)	(0.018)	(0.004)	(0.001)	(0.004)	(0.000)	(0.001)	(0.002)
May	-0.004	-0.052*	-0.017	-0.010	-0.011	-0.005	-0.005	-0.006	-0.006*	0.003	0.000	0.000
	(0.005)	(0.028)	(0.017)	(0.011)	(0.016)	(0.020)	(0.004)	(0.004)	(0.003)	(0.002)	(0.002)	(0.002)
Jun	0.004	0.041	-0.039***	0.017	-0.001	0.003	0.006**	-0.000	-0.000	-0.001	-0.000	0.000
	(0.005)	(0.038)	(0.008)	(0.014)	(0.011)	(0.010)	(0.003)	(0.003)	(0.003)	(0.001)	(0.001)	(0.001)
Jul	-0.001	-0.009	0.058**	0.012	-0.015	-0.001	-0.000	0.004	0.001	0.001	0.003	-0.001
	(0.006)	(0.022)	(0.023)	(0.007)	(0.019)	(0.019)	(0.005)	(0.005)	(0.003)	(0.002)	(0.002)	(0.002)
Aug	0.011	-0.006	-0.005	-0.012	0.030	0.056***	0.007***	-0.004	-0.001	-0.003	-0.002	-0.000
	(0.008)	(0.023)	(.)	(0.019)	(0.028)	(0.019)	(0.003)	(0.006)	(0.003)	(0.003)	(0.002)	(0.001)
Sep	-0.031***	0.030	0.034	-0.019**	0.015	-0.036*	-0.004	0.004	0.008*	-0.003	0.001	-0.004***
	(0.005)	(0.037)	(0.024)	(0.009)	(0.018)	(0.019)	(0.006)	(.)	(0.004)	(0.002)	(0.003)	(0.001)
Oct	0.013**	0.048	0.007	0.007	0.011	-0.040***	0.006	-0.001	-0.003	-0.001	0.001	0.000
	(0.005)	(0.043)	(0.022)	(0.013)	(0.025)	(0.010)	(0.005)	(0.004)	(0.003)	(0.002)	(0.002)	(0.002)
Nov	0.013	-0.005	-0.041*	0.015	-0.019	-0.006	-0.005	0.002	0.003	-0.001	0.004	-0.002
	(0.014)	(0.043)	(0.025)	(0.017)	(0.012)	(0.031)	(0.005)	(0.003)	(0.005)	(0.001)	(.)	(0.002)
Dec	0.006***	-0.003	-0.033	0.012	0.026	0.025	0.005	0.002	-0.002	-0.001	-0.002*	-0.000
	(0.002)	(0.033)	(0.031)	(0.016)	(.)	(0.022)	(0.003)	(0.006)	(0.002)	(0.001)	(0.001)	(0.001)
N	403	374	372	403	398	403	403	403	403	403	403	403
R^2	0.195	0.092	0.093	0.090	0.111	0.108	0.069	0.087	0.113	0.188	0.075	0.075

Notes: Observations not weighted for GDP in regressions. Lagged industry growth rate and monthly precipitation included. Regions 1 and 14, and regions 10 and 15 were merged for a total of 13 regions instead of 15 regions. Robust standard errors clustered by year and region in parentheses. ** p<0.01, ** p<0.05, * p<0.1
