# Supplementary material for: The impact of climate change on economic output across industries in Chile
Source: PLoS One. 2022 Apr 28;17(4):e0266811. doi: 10.1371/journal.pone.0266811 (PMC9049569; doi:10.1371/journal.pone.0266811)
Supplement: S3 Appendix — This appendix considers counterfactual exercises using the most recent “Shared Socioeconomic Pathways” (SSPs) scenarios published by the IPCC’s Sixth Assessment Report (IPCC 2021). (PDF) [file pone.0266811.s003.pdf]

S3 Appendix. Calibrated projections of climate change for Chile using the new IPCC (2021) SSP scenarios

This appendix considers the same counterfactual exercises described in Tables 7, 8 and 9, but using the most recent "Shared Socioeconomic Pathways" (SSPs) scenarios published by the IPCC's Sixth Assessment Report (IPCC 2021)(2). Tables 6, 7 and 8 considered the average temperature increase in the "Representative Concentration Pathways" (RCPs) available from IPCC Fifth Assessment Report (IPCC 2014)(1), describing different levels of greenhouse gases and other radiative forcings that might occur in the future. The RCPs described four pathways, spanning a broad range of forcing in 2100 (2.6, 4.5, 6.0, and 8.5 watts per meter squared), but purposefully did not include any socioeconomic "narratives" to go alongside them. The IPCC's Sixth Assessment Report (2) published five "Shared Socioeconomic Pathways" (SSPs) scenarios, which expand on the RCPs by also modelling how socioeconomic factors may change over the next century. These include things such as population, economic growth, education, urbanization, the rate of technological development and how different levels of climate change mitigation could be achieved when the mitigation targets of RCPs are combined with the SSPs.

Five SSP narratives describe alternative pathways for future society (2). Each SSP looks at how the different RCPs could be achieved within the context of the underlying socioeconomic characteristics and shared policy assumptions of that world. The SSPs five alternative socio-economic futures compromise: sustainable development (SSP1), middle-of-the-road development (SSP2), regional rivalry (SSP3), inequality (SSP4), and fossil-fuelled development (SSP5). SSP5-8.5 represents the high end of the range of future pathways, corresponding to RCP8.5. SSP3-7.0 lies between RCP6.0 and RCP8.5, and represents the medium to high end of the range of future forcing pathways. SSP4-6.0 corresponds to RCP6.0, fills in the range of medium forcing pathways. SSP2-4.5 represents the medium part of the range of future forcing pathways and updates RCP4.5. The SSPs are based on five narratives describing broad socioeconomic trends that could shape future society. These are intended to span the range of plausible futures. They include: a world of sustainability focused growth and equality (SSP1); a "middle of the road" world where trends broadly follow their historical patterns (SSP2); a fragmented world of "resurgent nationalism" (SSP3); a world of ever-increasing inequality (SSP4); and a world of rapid and unconstrained growth in economic output and energy use (SSP5).

Table C1, Table C2 and Table C3 have the same counterfactual exercise of Table 7, Table 8 and Table 9, respectively, but using the median temperature increase of the SSP1-2.6, SSP2-4.5, SSP3-6.0, SSP4-7.0 and SSP5-8.5, which consider increasingly worse scenarios for global warming. The simulated impacts are very similar to Tables 7, 8 and 9, although with slightly worse temperature scenarios.

Table C1 applies all the model's coefficients in the forecast. Under this assumption, Fishing, Mining, Home property and Public administration are the only industries hurt by climate change whether at the horizons of 2050 or 2100. In particular, Fishing's GDP almost disappears by 2100, even with just a 1.0°C increase in temperature. Mining and Home property would also decrease by at least 58% and 27%, respectively, by 2100. However, climate change would have a strong positive impact on the other economic sectors and therefore the total Chilean GDP would increase across all

scenarios in 2050 and 2100, which depends obviously on the unrealistic assumption that the coefficients are fixed over time.

Table C1. Simulated impact (in %) of the climate change on the industry and overall GDP level and growth rates in Chile for the period 1985-2017 and for the future (monthly model, all coefficients, Table 5)

Temperat. increase	(1)	(2)	(3)	(4)	(5)	(6)	(7)	(8)	(9)	(10)	(11)	(12)	Total GDP
Impact on GDP level in 2050 relative to no warming after 2017													
1.0C	24.7	-77.6	-27.6	53.0	38.1	22.6	7.0	3.5	20.6	-11.2	1.7	-3.3	9.8
1.3°C	33.3	-85.7	-34.3	73.8	52.2	30.4	9.2	4.5	27.5	-14.3	2.2	-4.3	14.0
1.4C	36.3	-87.7	-36.4	81.3	57.2	33.1	10.0	4.9	29.9	-15.3	2.4	-4.6	15.5
1.4°C	36.3	-87.7	-36.4	81.3	57.2	33.1	10.0	4.9	29.9	-15.3	2.4	-4.6	15.5
2.0°C	42.4	-90.9	-40.4	97.4	67.7	38.6	11.5	5.6	34.9	-17.3	2.8	-5.3	18.7
Impact on GDP level in 2100 relative to no warming after 2017													
1.1°C	82.3	-98.3	-58.4	217.4	140.6	74.1	20.3	9.7	66.2	-27.6	4.7	-8.8	42.4
2.0°C	198.0	-99.9	-79.7	716.6	393.3	174.0	39.9	18.3	151.9	-44.5	8.8	-15.5	132.4
2.4C	270.8	-100.0	-85.3	1142.9	578.8	235.2	49.7	22.3	203.1	-50.6	10.6	-18.3	202.9
2.9°C	387.1	-100.0	-90.1	2001.0	911.7	331.3	62.8	27.6	281.8	-57.4	13.0	-21.6	336.7
3.8°C	696.3	-100.0	-95.2	5305.5	1974.7	578.8	89.3	37.6	478.7	-67.3	17.3	-27.3	813.4

Notes: (1) Agriculture and Forestry, (2) Fishing, (3) Mining, (4) Manufacturing, (5) EGA, (6) Construction, (7) Commerce, Restaurants, and Hotels, (8) Transport and Communications, (9) Financial Services, (10) Home Ownership, (11) Personal Services, (12) Public Administration.

Table C2 applies only the model's coefficients that are statistically significant at the 10% level at least. Under this assumption, Agriculture, Fishing, Manufacture, EGA, Construction and Commerce are the only industries hurt by climate change whether at the horizons of 2050 or 2100. Even with just a 1.1°C increase in temperature, Agriculture, Fishing, Manufacture, EGA, Construction and Commerce would decline by 75%, 99.6%, 63.8%, 83.5%, 12.9% and 27.6%, respectively, around 2100. However, climate change would have a strong positive impact on the other economic sectors and therefore the total Chilean GDP would change only slightly in 2050 and it would even increase across all scenarios in 2100. Again, however, this result is strongly dependent on the positive effects of climate change estimated for some sectors and these effects may not materialize, since such positive effects may decline over time and even turn into negative effects.

Table C2. Simulated impact (in %) of the climate change on the industry and overall GDP level and growth rates in Chile:Only statistically significant coefficients (monthly model, Table 5)

Temperat. increase	(1)	(2)	(3)	(4)	(5)	(6)	(7)	(8)	(9)	(10)	(11)	(12)	Total GDP
Impact on GDP level in 2050 relative to no warming after 2017 (RCP 2.6, 4.5, 6.0, 8.5)													
1.0°C	-40.0	-87.2	8.9	-31.2	-48.5	-5.0	-11.2	0.0	33.5	0.0	3.5	0.0	-2.3
1.3°C	-48.5	-93.1	11.7	-38.5	-57.8	-6.4	-14.3	0.0	45.6	0.0	4.5	0.0	-1.9
1.4°C	-51.0	-94.4	12.6	-40.8	-60.5	-6.9	-15.3	0.0	49.9	0.0	4.9	0.0	-1.7
1.4°C	-51.0	-94.4	12.6	-40.8	-60.5	-6.9	-15.3	0.0	49.9	0.0	4.9	0.0	-1.7
2.0°C	-55.8	-96.3	14.6	-45.0	-65.4	-7.8	-17.3	0.0	58.8	0.0	5.6	0.0	-1.2
Impact on GDP level in 2100 relative to no warming after 2017 (RCP 2.6, 4.5, 6.0, 8.5)													
1.1°C	-75.0	-99.6	26.0	-63.8	-83.5	-12.9	-27.6	0.0	119.3	0.0	9.7	0.0	5.0
2.0°C	-92.0	-100.0	52.2	-84.2	-96.2	-22.3	-44.5	0.0	317.0	0.0	18.3	0.0	33.9
2.4°C	-95.1	-100.0	65.5	-89.1	-98.0	-26.1	-50.6	0.0	454.9	0.0	22.3	0.0	55.8
2.9°C	-97.4	-100.0	83.9	-93.1	-99.1	-30.6	-57.4	0.0	693.0	0.0	27.6	0.0	94.2
3.8°C	-99.2	-100.0	122.1	-97.0	-99.8	-38.0	-67.3	0.0	1407.7	0.0	37.6	0.0	210.0

Notes: (1) Agriculture and Forestry, (2) Fishing, (3) Mining, (4) Manufacturing, (5) EGA, (6) Construction, (7) Commerce, Restaurants, and Hotels, (8) Transport and Communications, (9) Financial Services, (10) Home Ownership, (11) Personal Services, (12) Public Administration.

Table C3 uses only the model's coefficients that are both negative and statistically significant. Again, under this assumption, Agriculture, Fishing, Manufacture, EGA, Construction and Commerce are the only industries hurt by climate change whether at the horizons of 2050 or 2100. In terms of the negative impact of climate change on the total GDP, it could range between 9% and 12.7% in 2050 and between 17.8% and 29.8% in 2100.

Table C3. Simulated impact (in %) of the climate change on the industry and overall GDP level and growth rates in Chile: Only statistically significant coefficients with a negative value (monthly model, Table 5)

Temperat. increase	(1)	(2)	(3)	(4)	(5)	(6)	(7)	(8)	(9)	(10)	(11)	(12)	Total GDP
Impact on GDP level in 2050 relative to no warming after 2017 (RCP 2.6, 4.5, 6.0, 8.5)													
1.0°C	-40.0	-87.2	0.0	-31.2	-48.5	-5.0	-11.2	0.0	0.0	0.0	0.0	0.0	-9.0
1.3°C	-48.5	-93.1	0.0	-38.5	-57.8	-6.4	-14.3	0.0	0.0	0.0	0.0	0.0	-10.9
1.4°C	-51.0	-94.4	0.0	-40.8	-60.5	-6.9	-15.3	0.0	0.0	0.0	0.0	0.0	-11.5
1.4°C	-51.0	-94.4	0.0	-40.8	-60.5	-6.9	-15.3	0.0	0.0	0.0	0.0	0.0	-11.5
2.0°C	-55.8	-96.3	0.0	-45.0	-65.4	-7.8	-17.3	0.0	0.0	0.0	0.0	0.0	-12.7
Impact on GDP level in 2100 relative to no warming after 2017 (RCP 2.6, 4.5, 6.0, 8.5)													
1.1°C	-75.0	-99.6	0.0	-63.8	-83.5	-12.9	-27.6	0.0	0.0	0.0	0.0	0.0	-17.8
2.0°C	-92.0	-100.0	0.0	-84.2	-96.2	-22.3	-44.5	0.0	0.0	0.0	0.0	0.0	-24.0
2.4°C	-95.1	-100.0	0.0	-89.1	-98.0	-26.1	-50.6	0.0	0.0	0.0	0.0	0.0	-25.7
2.9°C	-97.4	-100.0	0.0	-93.1	-99.1	-30.6	-57.4	0.0	0.0	0.0	0.0	0.0	-27.5
3.8°C	-99.2	-100.0	0.0	-97.0	-99.8	-38.0	-67.3	0.0	0.0	0.0	0.0	0.0	-29.8

Notes: (1) Agriculture and Forestry, (2) Fishing, (3) Mining, (4) Manufacturing, (5) EGA, (6) Construction, (7) Commerce, Restaurants, and Hotels, (8) Transport and Communications, (9) Financial Services, (10) Home Ownership, (11) Personal Services, (12) Public Administration.

Therefore our model predicts a large and positive impact of climate change on the total Chilean GDP if one uses all the model's coefficients both in 2050 and 2100, a small impact of climate change in 2050 if the forecasts use just the statistically significant coefficients, and a moderately negative impact of climate change both in 2050 and 2100 if the forecasts apply just the negative and statistically significant coefficients. It is possible that the forecasts using all the model's coefficients are way too optimistic,

while the forecasts with just the negative and statistically significant coefficients can be too pessimistic since the coefficients are selected to clearly present a negative scenario.

Since the impact coefficients on each industry are more precisely estimated from USA data, we also implementing a counterfactual exercise by applying the industry coefficients from Table A21 in (3). This exercise in Table C4 shows its strongest impact in 2050 on Construction, which would decline between 12.7% and 19.6%. Home property, Financial Services and Manufactures would also be strongly hit, declining between 9.1% and 15.9% relative to a scenario with no climate change. However, due to the positive coefficient estimated for the Mining industry, the impact of climate change on the Chilean GDP in 2050 would be limited to a decline of 0.8% or less. In 2100 the projections for climate change's impact on total GDP would again become very positive, because the counterfactual would assume that the log-growth of Mining would add up linearly over time.

Table C4. Simulated impact (in %) of the climate change on the industry and overall GDP level and growth rates in Chile for the future*

Temperat. increase	(1)	(2)	(3)	(4)	(5)	(6)	(7)	(8)	(9)	(10)	(11)	(12)	Total GDP
Impact on GDP level in 2050 relative to no warming after 2017 (RCP 2.6, 4.5, 6.0, 8.5)													
1.0°C	-4.6	-4.6	53.4	-9.1	7.0	-12.7	-6.2	0.2	-10.3	-10.3	-7.7	-5.6	-0.8
1.3°C	-5.9	-5.9	74.4	-11.7	9.1	-16.2	-8.0	0.3	-13.2	-13.2	-9.9	-7.2	-0.4
1.4°C	-6.3	-6.3	82.0	-12.5	9.9	-17.3	-8.6	0.3	-14.1	-14.1	-10.6	-7.7	-0.2
1.4°C	-6.3	-6.3	82.0	-12.5	9.9	-17.3	-8.6	0.3	-14.1	-14.1	-10.6	-7.7	-0.2
2.0°C	-7.2	-7.2	98.2	-14.2	11.4	-19.6	-9.7	0.4	-15.9	-15.9	-12.0	-8.8	0.4
Impact on GDP level in 2100 relative to no warming after 2017 (RCP 2.6, 4.5, 6.0, 8.5)													
1.1°C	-11.9	-11.9	219.8	-22.9	20.1	-30.9	-16.0	0.6	-25.5	-25.5	-19.6	-14.5	7.1
2.0°C	-20.6	-20.6	727.7	-37.6	39.5	-48.9	-27.1	1.1	-41.5	-41.5	-32.7	-24.7	50.7
2.4°C	-24.1	-24.1	1163.1	-43.2	49.1	-55.4	-31.6	1.3	-47.4	-47.4	-37.9	-28.9	93.0
2.9°C	-28.4	-28.4	2042.3	-49.6	62.0	-62.3	-36.8	1.6	-54.0	-54.0	-43.7	-33.7	181.9
3.8°C	-35.4	-35.4	5445.3	-59.2	88.1	-72.1	-45.2	2.1	-63.9	-63.9	-52.9	-41.7	535.9

Notes: *Climate change temperature coefficients estimated for the USA industry (post-1997) from Table A21 (both years) in Colacito, Hoffmann and Phan (2019) (1) Agriculture and Forestry, (2) Fishing, (3) Mining, (4) Manufacturing, (5) EGA, (6) Construction, (7) Commerce, Restaurants, and Hotels, (8) Transport and Communications, (9) Financial Services, (10) Home Ownership, (11) Personal Services, (12) Public Admininistration.

Table C5 shows a very similar counterfactual exercise with impact coefficients for temperature estimated for the US, but it considers only the statistically significant coefficients from Table A21 in (3). The exercise also applies the Agriculture-Fishing and Manufacturing industries coefficients from Table A20 in (3), since those coefficients were estimated with a smaller standard-error, perhaps due to the higher importance of such industries for the US economy before 1997. The results show a negative impact of climate change for most industries, except for Mining, Energy-Gas-Water (EGA) and Transports-Communications. The strongest impact of climate change is now estimated to be for the Agriculture and Fishing sectors, followed by Construction, Financial services and Home property. In particular, Agriculture and Fishing may decline between 18.7% and 28.2% in 2050, relative to a scenario with no additional climate change. Construction, Financial services and Home property would decline between 10.3% and 19.6% in 2050 due to the worsening climate change. In terms of the total GDP, the effect of climate change in 2050 would imply a deterioration between 6.8% and 10.5%. By 2100 the Agriculture and Fishing industries would decline between 43% and 85.7%, while the Construction, Financial services and Home property would decline between 25.5% and 72.1% due to climate change. Climate change by 2100 could imply a deterioration between 16.9% and 43% of the total Chilean GDP.

Table C5. Simulated impact (in %) of the climate change on the industry and overall GDP level and growth rates in Chile for the future*

Temperat. increase	(1)	(2)	(3)	(4)	(5)	(6)	(7)	(8)	(9)	(10)	(11)	(12)	Total GDP
Impact on GDP level in 2050 relative to no warming after 2017 (RCP 2.6, 4.5, 6.0, 8.5)													
1.0°C	-18.7	-18.7	0.0	-4.6	0.0	-12.7	-6.2	0.0	-10.3	-10.3	-7.7	-5.6	-6.8
1.3°C	-23.6	-23.6	0.0	-5.9	0.0	-16.2	-8.0	0.0	-13.2	-13.2	-9.9	-7.2	-8.7
1.4°C	-25.2	-25.2	0.0	-6.3	0.0	-17.3	-8.6	0.0	-14.1	-14.1	-10.6	-7.7	-9.3
1.4°C	-25.2	-25.2	0.0	-6.3	0.0	-17.3	-8.6	0.0	-14.1	-14.1	-10.6	-7.7	-9.3
2.0°C	-28.2	-28.2	0.0	-7.2	0.0	-19.6	-9.7	0.0	-15.9	-15.9	-12.0	-8.8	-10.5
Impact on GDP level in 2100 relative to no warming after 2017 (RCP 2.6, 4.5, 6.0, 8.5)													
1.1°C	-43.0	-43.0	0.0	-11.9	0.0	-30.9	-16.0	0.0	-25.5	-25.5	-19.6	-14.5	-16.9
2.0°C	-64.1	-64.1	0.0	-20.6	0.0	-48.9	-27.1	0.0	-41.5	-41.5	-32.7	-24.7	-27.6
2.4°C	-70.7	-70.7	0.0	-24.1	0.0	-55.4	-31.6	0.0	-47.4	-47.4	-37.9	-28.9	-31.6
2.9°C	-77.3	-77.3	0.0	-28.4	0.0	-62.3	-36.8	0.0	-54.0	-54.0	-43.7	-33.7	-36.1
3.8°C	-85.7	-85.7	0.0	-35.4	0.0	-72.1	-45.2	0.0	-63.9	-63.9	-52.9	-41.7	-43.0

Notes: *Climate change temperature coefficients estimated for the USA industry (post-1997) from Table A21 (both years in Colacito, Hoffmann and Phan (2019). (1) Agriculture and Forestry, (2) Fishing, (3) Mining, (4) Manufacturing, (5) EGA, (6) Construction, (7) Commerce, Restaurants, and Hotels, (8) Transport and Communications, (9) Financial Services, (10) Home Ownership, (11) Personal Services, (12) Public Administration.

References

1. IPCC (2014). "AR5 Scenario Database". `https://tntcat.iiasa.ac.at/AR5DB/`.

2. IPCC (2021). "IPCC Working Group I (WGI): Sixth Assessment Report". `https://interactive-atlas.ipcc.ch/`.

3. Colacito, R., B. Hoffmann and T. Phan (2019). "Temperature and Growth: A Panel Analysis of the United States". *Journal of Money, Credit and Banking*, 51(2-3), 313-368.
