# Supplementary material for: The impact of climate change on economic output across industries in Chile
Source: PLoS One. 2022 Apr 28;17(4):e0266811. doi: 10.1371/journal.pone.0266811 (PMC9049569; doi:10.1371/journal.pone.0266811)

S4 Appendix. Precipitation and temperature evolution statistics between 1950 and 2017

This appendix shows the results of the yearly temperature and precipitation fluctuations by macrozone weighted by the GDP of each region (instead of surface area, since some regions are large in area, but with little population and economic activity). To summarize the regional heterogeneity in a more succinct way, we create 4 macrozones, with macrozone 1 ”North Chile” corresponding to regions I, II, III, IV and XV, macrozone 2 ”Central Chile” corresponding to regions V, VI, VII, VIII, macrozone 3 ”South Chile” corresponding to regions IX, X, XI, XII and XIV, and macrozone 4 ”Metropolitan Region” corresponding to region XIII (which concentrates around 45% of the population and GDP of the nation). Figure 3 shows the yearly precipitation and temperature for each macrozone between 1950 and 2017 with weighted values according to the GDP of each region in 2017. Figure 3 shows that mean precipitation has been falling in the Central, South and Metropolitan macrozones, while mean temperatures have been increasing across all the macrozones. These patterns are qualitatively similar to the results weighted by surface area in Figure 2 of this article.

Fig D 1. The evolution of the yearly precipitation and temperature (weighted by the regional GDP in 2017) for each macrozone during the period 1950-2017. Minimum, Maximum and Mean values are from January to December of each year.

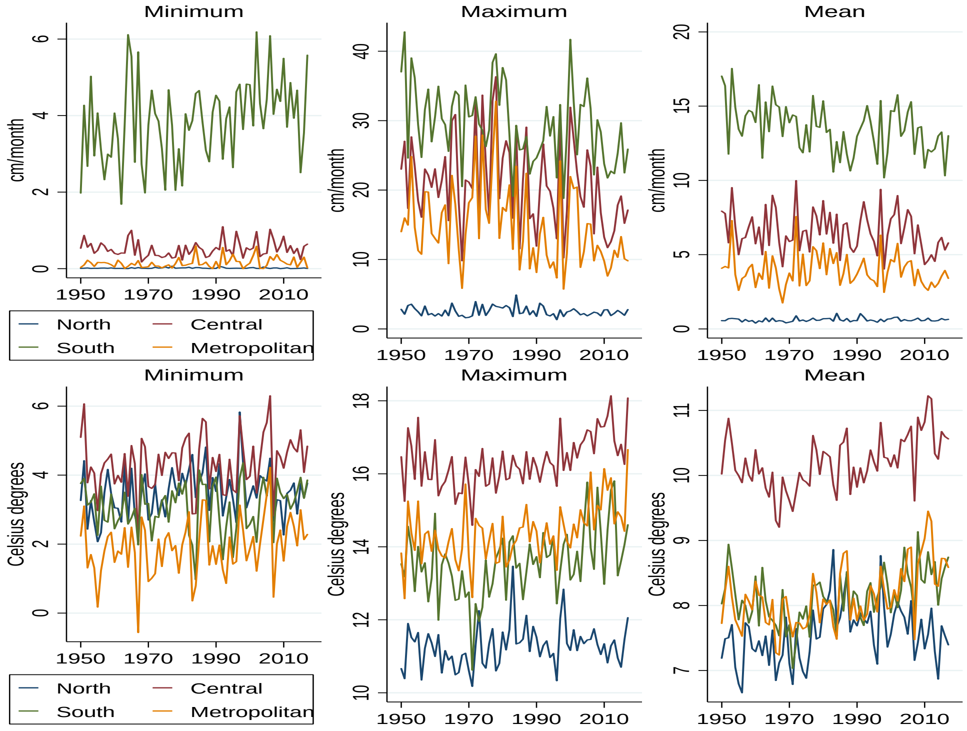

Supplement: S4 Appendix — This appendix shows the results of the yearly temperature and precipitation fluctuations by macrozone weighted by the GDP of each region. (PDF) [file pone.0266811.s004.pdf]
