# Supplementary material for: The impact of climate change on economic output across industries in Chile
Source: PLoS One. 2022 Apr 28;17(4):e0266811. doi: 10.1371/journal.pone.0266811 (PMC9049569; doi:10.1371/journal.pone.0266811)

S5 Appendix. GDP across regions

Figure E1 shows the share of the GDP (in %) across regions in Chile, according to the averages between 1985 and 2017. Figure E2 shows the same values of regional GDP in the most recent year of 2017, confirming that there was no difference in terms of the economic importance of each region in recent years.

Fig E 1. Share of the GDP (in %) across regions in Chile, according to the averages between 1985 and 2017

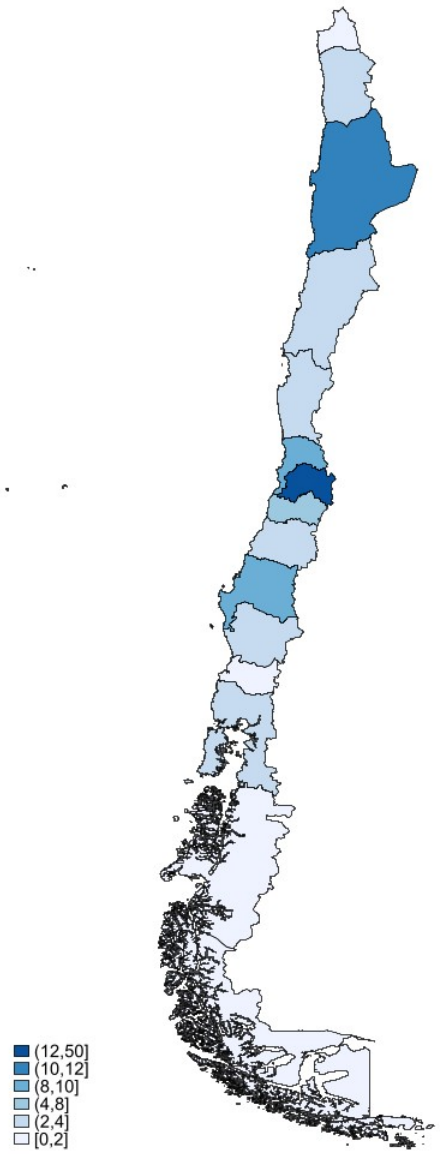

Fig E 2. Share of the GDP (in %) across regions in Chile, according to the values in 2017

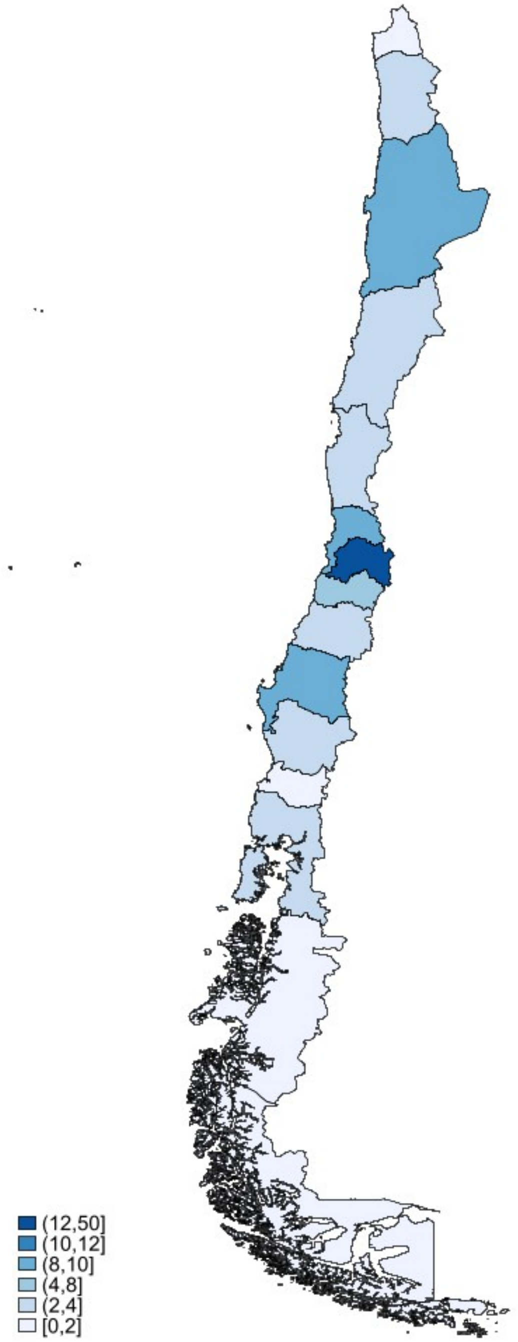

Supplement: S5 Appendix — (PDF) [file pone.0266811.s005.pdf]
