# Supplementary material for: The impact of climate change on economic output across industries in Chile
Source: PLoS One. 2022 Apr 28;17(4):e0266811. doi: 10.1371/journal.pone.0266811 (PMC9049569; doi:10.1371/journal.pone.0266811)
Supplement: Attachment — Submitted filename: Response to Editor and Journal requirements PONE-D-21-33259.pdf [file pone.0266811.s007.pdf]

Reply to the Editor and Journal requirements on "The impact of climate change on economic output across industries in Chile" PONE-D-21-33259

Dear Editor Francisco Aguilar and Plos One journal office,

Thank you for your report, suggestions and the two anonymous reviewers' feedback reports.

Requirement 1: When submitting your revision, we need you to address these additional requirements.

Please ensure that your manuscript meets PLOS ONE's style requirements, including those for file naming. The PLOS ONE style templates can be found at

https://journals.plos.org/plosone/s/file?id=wjVg/PLOSOne_formatting_sample_main_body.pdf and

https://journals.plos.org/plosone/s/file?id=ba62/PLOSOne_formatting_sample_title_authors_affiliation

Reply: We now send the manuscript with the Plos One template and the correct author affiliations.

Requirement 2: Please update your submission to use the PLOS LaTeX template. The template and more information on our requirements for LaTeX submissions can be found at http://journals.plos.org/plosone/s/latex.

Reply: We now send the manuscript with the Plos One template.

Requirement 3: We note that you have stated that you will provide repository information for your data at acceptance. Should your manuscript be accepted for publication, we will hold it until you provide the relevant accession numbers or DOIs necessary to access your data. If you wish to make changes to your Data Availability statement, please describe these changes in your cover letter and we will update your Data Availability statement to reflect the information you provide.

Reply: We published the entire dataset in Stata (.dta) format online on the Mendeley repository:

Madeira, C. (2022), "Panel data for the 15 Chilean regions with Weather and GDP variables", *Mendeley Data*, V1, doi: 10.17632/zyrdg56hzr.1.

Requirement 4: Please amend your list of authors on the manuscript to ensure that each author is linked to an affiliation. Authors' affiliations should reflect the institution where the work was done (if authors moved subsequently, you can also list the new affiliation stating "current affiliation:. . . ." as necessary).

Reply: We now send the manuscript with the correct author affiliations.

Additional Editor Comments:

1) Two reviewers have offered constructive criticisms to the submitted manuscript, all of which shall be fully addressed. For instance, the authors should clearly state the contribution of this study and the rationale supporting their model specification. Within their econometric estimation, a justification for not using common approaches to panel data (e.g. fixed, random, mixed effects) is necessary. Preferably, and if relevant, such models should be run. The model seems to be later calibrated with US-based data, which should be properly motivated and explained within a Methods section.

Reply: We justify the calibration with US-based data in the appendix, because the US is at the technological frontier and therefore may represent a better calibration for the future of Chile. Furthermore, the US has 50 states and longer panel data time series, therefore its calibration for the parameters can be less noisy than the shorter time-series and smaller regions of the Chilean dataset. This is justified in section 3.4 of the article. It is worth noting that the methodology applied for the US data by Colacito et al. (2019) is the same exact methodology we applied for Chile, therefore there is no methodological difference. Furthermore, the results with the US calibration are very similar and these are meant just as a robustness check to the main results.

2) Editorial observations: Section 2 should be re-titled to 'Methods' or 'Methods and Data'. Other subtitles such as 'Results' should be simply labeled as such (the Results are for Chile as clearly stated from the Title, so there is no need to include 'Chile' in a subtitle). The language is adequate but some careful editing is needed. As a case in point, the authors write: "The analysis for the past 35 years would show that 85% of the economic activity..." In this case "past" should be avoided as there will likely be a mismatch between time of publication and the period covered in the study. The word "would" is not needed, as their econometrics results indeed show these trends. A revised text could read" Our analysis over the 1985-2017 period show that 85% of the economic activity...". Although this might seem trivial, it will help with readability and possible increase the impact of the manuscript.

[Note: HTML markup is below. Please do not edit.]

Reviewers' comments:

Reviewer's Responses to Questions

Reply: We followed your suggestion and checked again the manuscript for typos.

Comments to the Author

1. Is the manuscript technically sound, and do the data support the conclusions?

The manuscript must describe a technically sound piece of scientific research with data that supports the conclusions. Experiments must have been conducted rigorously, with appropriate controls, replication, and sample sizes. The conclusions must be drawn appropriately based on the data presented.

Reviewer #1: Partly

Reviewer #2: Yes

Reply: We extended the manuscript with 6 new Tables and 5 new Figures plus additional analysis to support the conclusions.

2. Has the statistical analysis been performed appropriately and rigorously?

Reviewer #1: No

Reviewer #2: Yes

Reply: We added clarifying notes on the Introduction and section 2.3. We also added Tables 3, A1, A2, B4, B5, B6 and Figures 1, 2, 3, 4, 5, with additional statistical analysis.

3. Have the authors made all data underlying the findings in their manuscript fully available?

The PLOS Data policy requires authors to make all data underlying the findings described in their manuscript fully available without restriction, with rare exception (please refer to the Data Availability Statement in the manuscript PDF file). The data should be provided as part of the manuscript or its supporting information, or deposited to a public repository. For example, in addition to summary statistics, the data points behind means, medians and variance measures should be available. If there are restrictions on publicly sharing data—e.g. participant privacy or use of data from a third party—those must be specified.

Reviewer #1: No

Reviewer #2: Yes

Reply: We published the entire dataset in Stata (.dta) format online on the Mendeley repository:

Madeira, C. (2022), "Panel data for the 15 Chilean regions with Weather and GDP variables", *Mendeley Data*, V1, doi: 10.17632/zyrdg56hzr.1.

4. Is the manuscript presented in an intelligible fashion and written in standard English?

PLOS ONE does not copyedit accepted manuscripts, so the language in submitted articles must be clear, correct, and unambiguous. Any typographical or grammatical errors should be corrected at revision, so please note any specific errors here.

Reviewer #1: Yes

Reviewer #2: Yes

Reply: We reviewed again the manuscript for typos.

5. Review Comments to the Author

Please use the space provided to explain your answers to the questions above. You may also include additional comments for the author, including concerns about dual publication, research ethics, or publication ethics. (Please upload your review as an attachment if it exceeds 20,000 characters)

Reply: We provide two files with detailed replies to each reviewer.

We formatted the article according to the requirements of the editor and the journal's office. We hope you are pleased and that our article is now ready to be accepted by the *PLOS ONE*. Kind regards,

Karla Hernández
Carlos Madeira
