# Supplementary material for: The impact of climate change on economic output across industries in Chile
Source: PLoS One. 2022 Apr 28;17(4):e0266811. doi: 10.1371/journal.pone.0266811 (PMC9049569; doi:10.1371/journal.pone.0266811)
Supplement: Attachment — Submitted filename: review_PONE-D-21-33259.docx [file pone.0266811.s006.docx]

The authors in this manuscript investigate the impact of precipitation changes and temperature fluctuations over the period 1985-2017 in the Chilean case over 12 economic sectors. The paper is well written and despite several models offered to the reader, is able to provide straight conclusions. Nonetheless, I believe the manuscript could be further enhanced with the following suggestion listed below.

When the authors describe the data and where they retrieved them, they just mention the source (e.g., Central Bank of Chile, Chilean Bureau of Official Statistics, University of Delaware Air Temperature and Precipitation) without providing an effective reference in the references list. For completeness also these data sources should be properly mentioned in the references list. In this case also the effective date when data has been retrieved should be reported but I guess it could be not necessary.

The structure of your paper for sure is not “classical”, at least for my experience since an effective section for the literature review is missing while it is integrated within the Introduction section. Personally, this does not represent an issue as long as the Editor approves this structure.

There is a quite interesting study of the Standford University [1] which could be interested, and I guess it is worth mentioning, especially for either the Introduction or Conclusion section of your manuscript.

When you present the econometric model in section 2.3 you may add for completeness a classic reference for panel data models, such as the book of Baltagi [2].

You did not provide any preliminary analysis of your data, such as presence of autocorrelation, heterogeneity, cross-sectional dependency, or stationarity of your series. Since in your model you analyze GDP growth you should not have problems in terms of unit roots. Nonetheless, these issues are often undervalued in panel data analysis.

In your panel model you used robust standard errors clustered by region and year. However, are robust to which disturbance? I guess they are robust to heteroscedasticity and autocorrelation. Nonetheless, since you are working with regional data, maybe the sample could be affected also by cross-sectional dependency. This is an issue which could be addressed with time fixed effects or, for example, using proper robust standard errors able to take into account this disturbance. You may be interested in giving a look to Driskoll and Kraay robust standard errors, for example [3-4].

You performed your econometric model with both quarterly and monthly data. Since region-industry data is available only with yearly frequency, have you attempted to perform your model also with just yearly average data of temperature and precipitations?

Since you are dealing with regional data for Chile, maybe it could be an idea to enhance your manuscript also with a graphical regional representation of how each Chilean region – i.e., a choropleth map – contribute to the composition of the entire GDP of the country (considering a specific year of interest or an average of the time-range of your analysis) or for some specific sectors of interest (maybe you may add in the appendix the maps for all remaining sectors).

In the concluding section of your manuscript, you may consider some possible extension of your analysis, for example through the use of spatial [5-7] or dynamic time-series panel data models [8-9].

[1] Diffenbaugh, N. S., & Burke, M. (2019). Global warming has increased global economic inequality. *Proceedings of the National Academy of Sciences, 116*(20), 9808-9813.

[2] Baltagi, B. H. (2021). *Econometric analysis of panel data*. Springer Nature.

[3] Driscoll, J. C., & Kraay, A. C. (1998). Consistent covariance matrix estimation with spatially dependent panel data. *Review of economics and statistics, 80*(4), 549-560.

[4] Hoechle, D. (2007). Robust standard errors for panel regressions with cross-sectional dependence. *The Stata Journal, 7*(3), 281-312.

[5] Millo, G., & Piras, G. (2012). splm: Spatial panel data models in R. *Journal of statistical software, 47*, 1-38.

[6] Elhorst, J. P. (2014). Spatial panel data models. In *Spatial econometrics* (pp. 37-93). Springer.

[7] Belotti, F., Hughes, G., & Mortari, A. P. (2017). Spatial panel-data models using Stata. *The Stata Journal*, *17*(1), 139-180.

[8] Chudik, A., Mohaddes, K., Pesaran, M. H., & Raissi, M. (2013). Debt, inflation and growth: robust estimation of long-run effects in dynamic panel data models. Cafe research paper, (13.23).

[9] Chudik, A., Mohaddes, K., Pesaran, M. H., & Raissi, M. (2018). Rising public debt to GDP can harm economic growth. Economic Letter, 13(3), 1-4.
